# Bakuchiol Ferulate: A Novel Functional Retinol Analog With Enhanced Photostability and Reduced Phototoxicity for Cosmetic Applications

**DOI:** 10.1111/jocd.71067

**Published:** 2026-07-16

**Authors:** Jiangming Zhong, Yizhen Yan, Nan Zhao, Xinyu Cao, Yuting Liang, Qi Zhou, Jinjing Bao, Zhiwei Li, Qiaoyuan Liu, Meixin Lu, Qing Liu, Bin Shuai, Rong Hu, Peng Shu

**Affiliations:** ^1^ HBN Research Institute and Biological Laboratory Shenzhen Hujia Technology Co. Ltd. Shenzhen Guangdong China; ^2^ Guangdong Engineering Technology Research Center for Functional Skincare Innovation Shenzhen Hujia Technology Co. Ltd. Shenzhen Guangdong China

**Keywords:** bakuchiol ferulate, molecular hybridization, photostability, phototoxicity, retinoid‐like activity, transcriptome profiling

## Abstract

**Background:**

Retinol (ROL) is essential in dermatology for antiaging; however, its clinical utility is frequently limited by inherent chemical instability and significant cutaneous irritation.

**Objective:**

This study describes the development of bakuchiol ferulate (BF), a novel molecular hybrid synthesized from bakuchiol and a ferulic acid (FA) analog. The study aimed to evaluate BF as a stable, well‐tolerated, and efficacious alternative to ROL.

**Methods:**

The performance of BF was compared against ROL, bakuchiol (BKU), and FA. Stability was assessed using HPLC‐based degradation assays and electron paramagnetic resonance (EPR) spectroscopy. Cytotoxicity was evaluated via CCK‐8 assays in HaCaT and 3T3 cell lines. Phototoxicity was determined using the 3T3‐NRU assay (OECD 432) and singlet oxygen/superoxide ions generation analysis. A 14‐day human cumulative irritation patch test was conducted to compare the dermal compatibility and Mean Cumulative Irritancy Index (MCII) of varying concentrations of BF and ROL. Finally, ROL‐like activity and molecular mechanisms were characterized through transcriptome profiling in human dermal fibroblasts.

**Results:**

BF demonstrated superior thermal and photostability compared to both ROL and BKU. In phototoxicity testing, BF exhibited the lowest photo‐reactivity and UV‐triggered ROS induction, maintaining a non‐phototoxic profile. Clinical results showed that all concentrations of BF remained nonirritating with negligible MCII scores, demonstrating significantly higher dermal tolerance in human subjects. Transcriptomic analysis revealed that BF's gene expression profile highly correlates with that of ROL, specifically upregulating pathways involved in the cell cycle and DNA repair. Crucially, BF achieved these effects while avoiding the pro‐apoptotic and inflammatory signatures typically associated with ROL treatment.

**Conclusion:**

BF functions as a potent functional ROL analog that successfully merges structural stability with minimized phototoxicity. These findings suggest that BF offers a stable, efficacious, and safer alternative for advanced cosmetic and dermatological applications.

## 1 Introduction

Retinoids are a class of compounds chemically derived from vitamin A, characterized by a lipophilic structure consisting of an isoprenoid chain linked to a beta‐ionone ring. Available in both natural and synthetic forms, they are widely used in dermatology and cosmetics. Among retinoids, retinol (ROL) is particularly valued in cosmetic formulations for its demonstrated efficacy in addressing acne, skin aging, and other skin conditions [1]. Despite the therapeutic benefits, the application of ROL is constrained by significant challenges: it is highly unstable, sensitive to light and temperature, and prone to degradation [2]. Furthermore, topical use often causes cutaneous irritation [3]. These limitations hinder the broader incorporation of ROL into cosmetic and pharmaceutical formulations. From a clinical perspective, overcoming the dual challenges of retinoid dermatitis and rapid photo‐degradation is vital for improving patient compliance and treatment outcomes in daily dermatological practice. Developing a highly stable, nonirritating ROL alternative would allow clinicians and formulators to deliver consistent therapeutic antiaging benefits, even for individuals with highly sensitive skin or compromised skin barriers who typically cannot tolerate traditional retinoids.

Bakuchiol (BKU) is a plant‐derived compound that has shown promising antiaging effects when applied topically. Emerging evidence suggests that BKU may act as a functional analog of retinoids, as both modulate similar gene expression pathways and bring about comparable improvements in photoaged skin [4]. In addition to these shared benefits, BKU offers several distinct advantages over ROL: it possesses a favorable safety profile with reduced irritancy, suitable for sensitive skin, and is highly compatible with a broad range of emollients and solubilizers, making it easier to formulate [5].

To overcome the inherent limitations of many active compounds, a common strategy in cosmetic science involves the synthesis of novel derivatives to improve stability and bioactivity. For example, 3‐O‐ethyl‐l‐ascorbic acid overcomes the instability of vitamin C. α‐tocopheryl ferulate synergizes the properties of vitamin E and ferulic acid (FA) for superior efficacy [6, 7]. Esterification of all‐trans retinoic acid (ATRA) to hyaluronan (HA) to form HA‐ATRA resulted in significantly improved long‐term stability and reduced pro‐inflammatory cytokine expression compared to ROL, providing another example of conjugated derivatives [8].

Building upon this concept, we hypothesized that this molecular hybridization would merge the ROL‐like activity and favorable stability of BKU with the potent antioxidant capacity of FA, potentially yielding a superior, well‐tolerated alternative to ROL. We designed and synthesized a novel ester, bakuchiol ferulate (BF) by esterification of BKU with (2E)‐3‐(1,3‐benzodioxol‐5‐yl)‐2‐propenoic acid, which is an analog of FA. To investigate this novel hybrid, we compared BF against ROL, BKU, and FA for key performance indicators: photostability, phototoxicity, cumulative skin irritancy potential, and gene expression profiles. Our findings suggest that BF is a highly stable compound that effectively mimics ROL's beneficial activity while minimizing the adverse effects.

## 2 Materials and Methods

### 2.1 Preparation of BF

**FIGURE 1:**
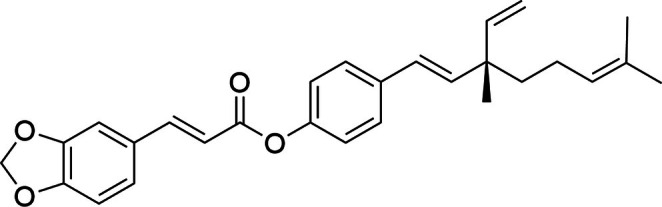
Chemical structure of Bakuchiol Ferulate.

The chemical structure of BF is shown in Figure 1. BF was synthesized via an esterification reaction. In a 50 mL brown round‐bottom flask, 230 mg of (2E)‐3‐(1,3‐benzodioxol‐5‐yl)‐2‐propenoic acid, 256 mg of BKU, 383 mg of 1‐ethyl‐3‐(3‐dimethylaminopropyl) carbodiimide (EDCI), and 12 mg of 4‐dimethylaminopyridine (DMAP) were added and dissolved in 5 mL of tetrahydrofuran (THF) under stirring at room temperature. Subsequently, 322 μL of triethylamine was added dropwise, and the reaction mixture was stirred at room temperature. The progress of the reaction was monitored by thin‐layer chromatography (TLC).

Upon completion, the reaction mixture was concentrated under reduced pressure and then dissolved in water. The aqueous phase was extracted three times with 25 mL portions of ethyl acetate. The combined organic extracts were dried over anhydrous sodium sulfate, filtered, and concentrated under reduced pressure to yield a crude product. The crude residue was purified by column chromatography using petroleum ether and ethyl acetate (10:1, v/v) as the eluent to afford BF as a white solid (329 mg, 77% yield). Compound BF was characterized using ^1^H ^13^C NMR spectroscopy. ^1^H NMR (400 MHz, CDCl_3_) *δ* 7.77 (d, *J* = 15.9 Hz, 1H), 7.39 (d, *J* = 8.6 Hz, 2H), 7.15–7.03 (m, 4H), 6.84 (d, *J* = 7.9 Hz, 1H), 6.44 (d, *J* = 15.8 Hz, 1H), 6.38–6.13 (m, 2H), 6.03 (s, 2H), 5.89 (dd, *J* = 17.5, 10.7 Hz, 1H), 5.16–4.99 (m, 3H), 1.97 (q, *J* = 7.5 Hz, 2H), 1.69 (s, 3H), 1.60 (s, 3H), 1.56–1.49 (m, 2H), 1.22 (s, 3H). ^13^C NMR (101 MHz, CDCl_3_) *δ* 165.59, 149.95, 149.71, 148.42, 146.20, 145.65, 138.07, 135.56, 131.33, 128.60, 126.94, 126.32, 124.86, 124.68, 121.59, 115.09, 112.08, 108.60, 106.56, 101.62, 42.62, 41.19, 25.66, 23.27, 23.19, 17.62. The ^1^H NMR and ^13^C NMR spectra are shown in Figures S1 and S2, respectively.

### 2.2 Light and Heat Stability Evaluation by HPLC

0.3% of BF, ROL, BKU (lipophilic) were dissolved in isononyl isononanoate, and 0.3% FA (hydrophilic) was dissolved in dipropylene glycol for stability evaluation. The testing solutions were stored in amber glass bottles with airtight closures. For stability assessment, samples were subjected to light exposure (5000 lx), dark storage, ambient temperature (25°C), and accelerated conditions (48°C) for 3 months. The stability of each compound was indicated by the residual quantitative analysis, which was assessed at Days 0, 7, 14, 28, 42, 56, 70, and 90. The quantitative analysis of different samples was performed using Vanquish Core high‐performance liquid chromatography (HPLC) (Thermo Fisher Scientific, US). Samples (0.5 g) were extracted via ultrasonication for 15 min with a methanol–THF (80:20, v/v) solvent system, filtered through a 0.22‐μm membrane, and analyzed under optimized chromatographic conditions: a C18 column (250 × 4.6 mm, 5 μm), isocratic elution with methanol–THF–water (90:5:5, v/v/v) at 1.0 mL/min, column temperature of 30°C, and detection at 330 nm. Quantification employed an external standard curve (0.5–50 μg/mL) prepared from a 1 mg/mL stock solution. Data were normalized to Day 0 (D0) values to calculate degradation kinetics.

### 2.3 Electron Paramagnetic Resonance (EPR) Spectroscopy

Superoxide anion (O_2_•^−^) was detected using a Bruker EMXplus‐10/12 spectrometer (Bruker, Germany). The test samples were dissolved in methanol containing 30% DMSO to obtain a final concentration of 10 mg/mL. Then, 20 μL of 5,5‐dimethyl‐1‐pyrroline *N*‐oxide (DMPO) was added to 1800 μL of the reaction mixture as a spin‐trapping agent. Samples were irradiated with a 100 W xenon lamp for 30 s to induce O_2_•^−^ generation. The characteristic signals of the DMPO–OOH adduct were analyzed by peak‐to‐peak intensity using Bruker Xenon software.

### 2.4 Cell Culture

The HaCaT cells and human dermal fibroblast (HDF) were obtained from Procell (Wuhan Pricella Biotechnology Co. Ltd.). The NIH/3T3 cells were obtained from the American Type Culture Collection (ATCC). The cells were cultured in high glucose Dulbecco's modified Eagle's medium (DMEM, Gibco) supplemented with 10% fetal bovine serum (FBS, Gibco) and maintained at 37°C in a humidified atmosphere containing 5% CO2.

### 2.5 Cell Viability Assay

HaCaT cells or NIH/3T3 cells were seeded in 96‐well plates and treated as indicated. Cell viability was assessed using a Cell Counting Kit‐8 (CCK‐8; Beyotime, C0043) according to the manufacturer's protocol. Following treatment, 10 μL of CCK‐8 solution was added to each well, and plates were incubated at 37°C for 2 h. Absorbance was measured at 450 nm with a reference wavelength of 650 nm using a microplate reader. Viability was expressed as a percentage relative to the untreated control group.

### 2.6 In Vitro Phototoxicity Assessment by Neutral Red Uptake (NRU) Assay

The phototoxic potential of the test substances was evaluated according to the OECD Test Guideline 432 using the BALB/c 3T3 fibroblast NRU assay [9]. BALB/c 3T3 cells were seeded in clear 96‐well plates at a density of 1 × 10^4^ cells per well in high‐glucose DMEM (Gibco) supplemented with 10% FBS (Gibco) and maintained at 37°C in a humidified 5% CO_2_ atmosphere. After 24 h, the wells were washed with PBS (Gibco, 20 012 027). Eight logarithmic dilutions of each test substance were prepared in assay medium (Table 1) and applied to the cells, followed by a 1‐h incubation. Two identical plates were prepared for each condition: one received UVA irradiation (+UV), while the other was kept in the dark (–UV). The +UV plates were irradiated with 5 J/cm^2^ UVA light using a UVA crosslinker (Luyor UCL‐3500) at an irradiance of approximately 7.2 mW/cm^2^ for 694.5 s. After irradiation or dark incubation, the test solutions were discarded, and the wells were washed with PBS. Cells were then incubated overnight in fresh DMEM. Subsequently, the medium was replaced with 50 μg/mL neutral red solution in DMEM and incubated for 3 h at 37°C. The neutral red solution was then removed, the cells were washed with PBS, and the incorporated dye was solubilized using a solution of acetic acid, water, and ethanol (1:49:50 v/v/v). Absorbance was measured at 540 nm using a microplate reader. The concentration causing 50% inhibition of viability (IC_50_) was determined for both –UV and +UV conditions using a four‐parameter logistic nonlinear regression model in GraphPad Prism (v6.0). The photoirritation factor (PIF) was calculated from these IC₅₀ values, and the results were classified based on the criteria outlined in Table 2.

**TABLE 1:** Test concentration gradients of test substance.

Concentration (μg/mL)	C1	C2	C3	C4	C5	C6	C7	C8
BF	12.5	25	50	100	200	400	600	800
ROL	12.5	25	50	100	200	400	600	800
BKU	7.5	15	31.25	62.5	125	250	500	1000
FA	7.5	15	31.25	62.5	125	250	500	1000
8‐MOP	6.25	12.5	25	50	100	150	200	250

**TABLE 2:** Prediction model for the in vitro 3T3‐NRU‐PT based on photoirritation factor (PIF) values.

PIF values	Prediction
PIF < 2	No phototoxicity
2 ≤ PIF < 5	Equivocal phototoxicity
PIF ≥ 5	Phototoxicity

### 2.7 Singlet Oxygen and Superoxide Ions Assay

HaCaT or 3T3 cells were seeded in black‐walled, clear‐bottom 96‐well plates at a density of 1 × 10^4^ cells per well and treated as indicated. To assess reactive oxygen species (ROS) generation, intracellular singlet oxygen and superoxide anion levels were measured using the Singlet Oxygen Sensor Green (SOSG) Assay Kit (Beyotime, S0068S) or the Superoxide Anion Detection Kit (Dihydroethidium, DHE; Beyotime, S0064S), respectively, according to the manufacturer's protocols.

After treatment application and 1 h of incubation, the cells were exposed to either UVA radiation (5 J/cm^2^) or UVB radiation (150 mJ/cm^2^). The test substances remained in contact with the cells for 0, 30, 60, 90, or 120 min postirradiation. The medium was then aspirated, and the cells were washed with PBS. Subsequently, SOSG or DHE working solution in culture medium was added and incubated for 20 min at 37°C. Fluorescence was measured using a microplate reader (Tecan Infinite E PLEX, Switzerland) at excitation/emission wavelengths of 504/525 nm for SOSG and 535/610 nm for DHE.

### 2.8 In Vitro Skin Permeation Assay

Percutaneous absorption was evaluated using excised Bama miniature pig skin (40–45 days old, ~1 mm thick) sourced in accordance with animal ethical standards. Skin integrity was verified, and four replicates were used per concentration of compound BF. The receptor compartment contained 8 mL of 100% methanol, equilibrated for 30–45 min at 32°C ± 1°C with magnetic stirring at 300 rpm. Following equilibration, 500 μL of test solution was applied to the donor compartment and sealed with Parafilm. Receptor fluid aliquots (1 mL) were collected at 1, 2, 4, 8, and 24 h.

Upon study termination, the donor compartment was rinsed eight times with 1 mL of methanol to recover residual compound. The stratum corneum was isolated via 10 sequential tape strips, which were extracted in 8 mL of methanol using ultrasonication. Remaining skin (1 cm^2^) was minced and similarly extracted at 25°C. All samples were centrifuged at 14 000 rpm for 15 min at 4°C, and the supernatants were filtered through a 0.22 μm nylon membrane prior to analysis.

### 2.9 Participants: Inclusion and Exclusion Criteria

All experimental procedures were conducted in strict accordance with the World Medical Association (WMA) Declaration of Helsinki and its subsequent amendments. Written informed consent was obtained from each participant prior to the commencement of any study‐related procedures. The study included healthy male and female volunteers aged 18–60 years. Subjects were excluded if they met any of the following criteria: (1) pregnancy or lactation; (2) a history of hypersensitivity; (3) insulin‐dependent diabetes mellitus; (4) immunodeficiency or autoimmune diseases; (5) history of bilateral mastectomy or bilateral axillary lymph node dissection; (6) active asthma or other chronic respiratory diseases requiring treatment; (7) receipt of anti‐cancer chemotherapy within the previous 6 months; (8) application of anti‐inflammatory drugs to the test site within the past 2 months; (9) use of antihistamines within the past week or immunosuppressants within the past month; (10) presence of unresolved inflammatory skin diseases; (11) the presence of scars, pigmentation, atrophy, port‐wine stains, or other blemishes at the test site that could interfere with clinical assessment; (12) concurrent participation in other clinical trials; or (13) nonvoluntary participation or inability to comply with the experimental protocol.

### 2.10 Cumulative Skin Irritation Test

Skin irritation potential was evaluated using a 14‐day cumulative patch test following the methodology described by Bowman et al. [10]. The study was conducted in a controlled environment (21°C ± 1°C, 50% ± 10% relative humidity). After signing informed consent, participants' volar forearms were cleaned and acclimated for 30 min. The test materials included varying concentrations of ROL (0.05%, 0.1%, and 0.3%), BF (0.05%, 0.1%, 0.3%, 0.5%, and 1.0%), and vehicle control. Each test material was applied randomly using square patch chambers (Beijing Baiyiyida Technology Development Co. Ltd.) to the volar forearm. Patches were applied daily for 23 ± 1 h over 14 consecutive days. During the study, participants were instructed to avoid washing the test area, swimming, strenuous exercise, and excessive sun exposure.

Skin reactions were visually assessed by blinded evaluators 30 min after each patch removal under standardized lighting, supplemented by VISIA‐CR (Canfield Scientific) imaging. Reactions were graded according to the International Contact Dermatitis Research Group (ICDRG) guidelines [11]:

− 0: Negative reaction.
− 1: Doubtful reaction (faint erythema/infiltration).
− 2: Erythema and infiltration covering the test area (+).
− 3: Strong erythema with many papules/vesicles (++).
− 4: Severe reaction with coalescing vesicles or bullae (+++).

To evaluate skin tolerance, the Cumulative Irritation Index (CII) was calculated for each test material and each participant according to established protocols [12, 13]. The CII was defined using the following formula:

$$\mathrm{CII}=\left(\text{ΣIrritation scores}\right)/\left(\text{Total number of readings}\right)$$

Baseline scores (Day 0) were excluded from this calculation. The Mean Cumulative Irritancy Index (MCII) for each test material was subsequently determined by averaging the individual CII values across all participants. The irritation potential of the test materials was categorized based on the calculated MCII values according to a four‐point classification scale [13]. Materials were classified as nonirritating (MCII < 0.025), slightly irritating (0.025 MCII < 1), moderately irritating (1 MCII < 2), or very irritating (2 MCII < 3).

### 2.11 RNA‐Seq and Bioinformatics Analysis

Total RNA was extracted from collected cells using TRIzol reagent (Invitrogen, USA), and RNA integrity was assessed with the Agilent 2100 Bioanalyzer. Polyadenylated mRNA was enriched from total RNA using the VAHTS mRNA Capture Beads Kit (Vazyme, China) and subsequently fragmented into 250–450 bp fragments. Sequencing libraries were prepared with the VAHTS Universal V8 RNA‐seq Library Prep Kit for Illumina (Vazyme, China) according to the manufacturer's protocol, and paired‐end sequencing was performed on an Illumina NovaSeq 6000 platform.

Raw sequencing reads were processed with Fastp (v0.23.2) to remove adapter sequences and low‐quality bases. Ribosomal RNA sequences were depleted using SortMeRNA (v4.3.4). High‐quality clean reads were aligned to the human reference genome (GRCh38) using STAR aligner (v2.7.10a). Gene expression levels were quantified, and differential expression analysis was conducted with DESeq2 (v1.34.0). Genes with an absolute log_2_ fold change greater than 1 and a *p* < 0.05 were considered significantly differentially expressed. Functional enrichment of differentially expressed genes was performed using the ClusterProfiler package in R, with KEGG pathway analysis to identify significantly enriched signaling pathways.

## 3 Results

### 3.1 Heat and Photodegradation Evaluation by HPLC

**FIGURE 2:**
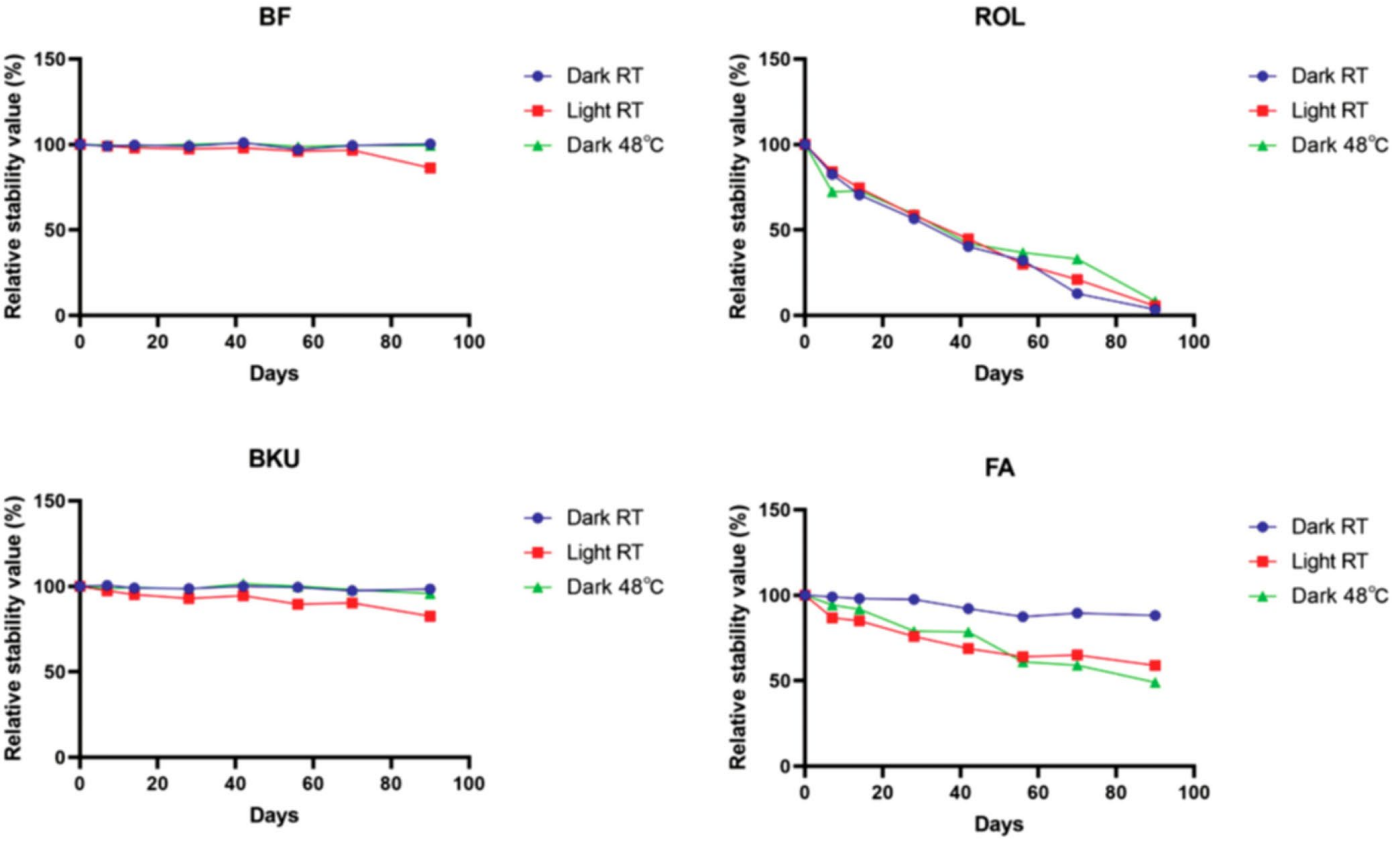
Chemical stability of BF, ROL, BKU, and FA under different storage conditions. The relative stability of each compound was monitored by HPLC over 90 days under three conditions: Dark at room temperature (blue), light exposure at room temperature (red), and dark at 48°C (green).

In accordance with the concentration limit for ROL established by the EU Scientific Committee on Consumer Safety (SCCS), test formulations were prepared at a concentration of 0.3% (w/w) for each compound (BF, ROL, BKU, and FA) [14]. The thermal and photostability of 0.3% BF, ROL, BKU, and FA were evaluated using HPLC over a 90‐day period to determine the percentage of each compound retained. As shown in Figure 2, under light exposure, a clear divergence was observed as early as Day 7, where ROL and FA retention fell below 84% and 86.8%, respectively, while BF remained stable at 99%. While BKU showed comparable stability to BF in the short term, it became susceptible to photodegradation after Day 56, eventually dropping to 82.58% by Day 90, compared to 86.32% for BF. At this point, ROL and FA only remained 5.55% and 58.98%, respectively.

Under thermal stress (48°C), the superiority of BF was even more pronounced. BF exhibited negligible degradation (99.42% retention) over 90 days. In contrast, FA and ROL demonstrated substantial thermal instability, retaining only 49.05% and 8.31%, respectively by the end of the study period. These data suggest that BF exhibits a synergistic stabilization effect that exceeds the performance of its individual precursors and ROL.

### 3.2 EPR Spectra Demonstrated BF With Least Photo‐Reactivity

**FIGURE 3:**
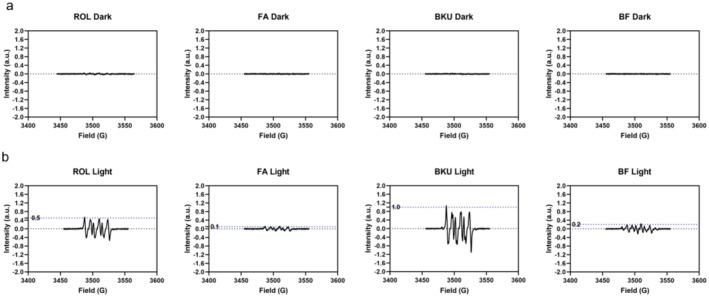
EPR spectra of (a‐b) ROL, FA, BKU, and BF under non‐illuminated or illuminated conditions.

The concentration of superoxide anion radicals produced by ROL, FA, BKU, and BF under non‐illuminated or illuminated conditions was detected and compared by EPR spectroscopy. As shown in Figure 3, it was found that under non‐illuminated conditions (a), the superoxide anion radical levels of the four raw materials were close to zero. After illumination (b), ROL and BKU would produce more radicals, while the radicals produced by FA and BF remained at a very low level, indicating that FA and BF have the least photo‐reactivity.

### 3.3 Cell Viability Assay Showed the Cytotoxicity of Tested Compounds

**FIGURE 4:**
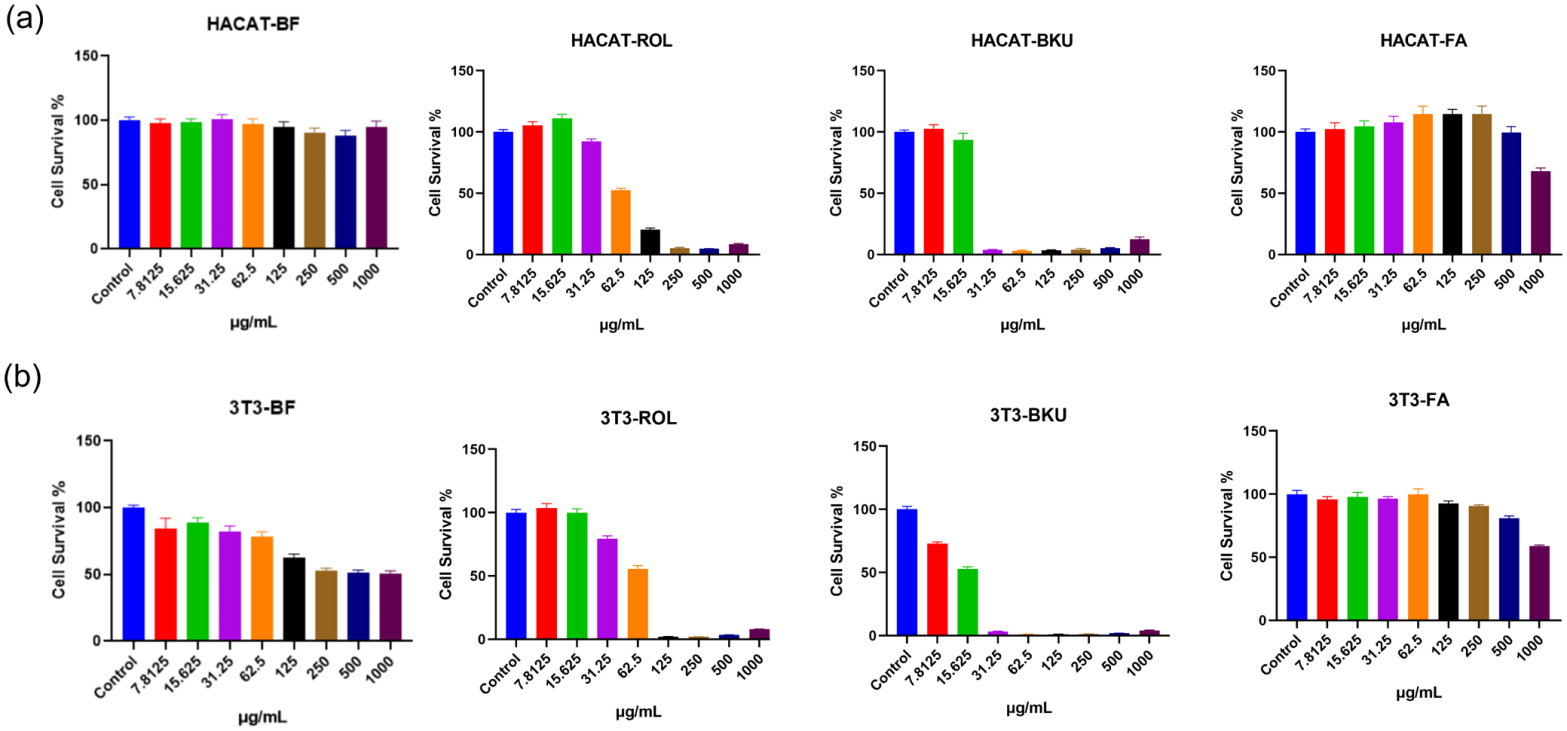
Cell viability assay for four compounds in (a) HaCaT and (b) 3T3 cell lines.

To evaluate the cytotoxicity of the four compounds, CCK‐8 assays were conducted in HaCaT and 3 T3 cell lines. Cells were treated with a concentration gradient of each compound (ranging from 7.8125 to 1000 μg/mL). Using 80% cell viability as the cytotoxicity threshold, as indicated in Figure 4, BKU exhibited the highest cytotoxicity, with viability falling below 80% at concentrations above 15.625 μg/mL in HaCaT cells and above 7.8 μg/mL in 3T3 cells. ROL showed no cytotoxic effects up to 31.25 μg/mL in HaCaT cells and 15.625 μg/mL in 3T3 cells. In contrast, BF demonstrated markedly lower cytotoxicity than both BKU and ROL, maintaining viability above 80% at concentrations up to 1000 μg/mL in HaCaT cells and 31.25 μg/mL in 3T3 cells. FA also displayed a favorable safety profile, showing 80% cell viability up to 500 μg/mL in both cell lines.

### 3.4 Assessment of Phototoxicity via 3T3‐NRU Assay

To evaluate the phototoxicity of BF compared to the others, the 3T3‐NRU phototoxicity assay was conducted according to the OECD Test Guideline 432. We introduced 8‐methoxypsoralen (8‐MOP) as a phototoxic positive control. The results demonstrated that 8‐MOP exhibited significant phototoxicity, with a PIF value of 25.07. The phototoxicity of compounds BF, ROL, BKU, and FA was further evaluated using the 3 T3‐NRU assay. As summarized in Table 3, the PIF values for BF, BKU, and FA were 1.14, 1.74, and 1, respectively, indicating no phototoxicity. ROL showed phototoxicity with a PIF value of 5.64.

**TABLE 3:** Results of OECD 432 assay.

Name	PIF	Conclusion
8‐MOP	25.07	Phototoxicity
BF	1.14	No phototoxicity
ROL	5.64	Phototoxicity
BKU	1.74	No phototoxicity
FA	1	No phototoxicity

### 3.5 Assessment of Phototoxic Potential via ROS Generation in UV‐Irradiated Skin Cells

**FIGURE 5:**
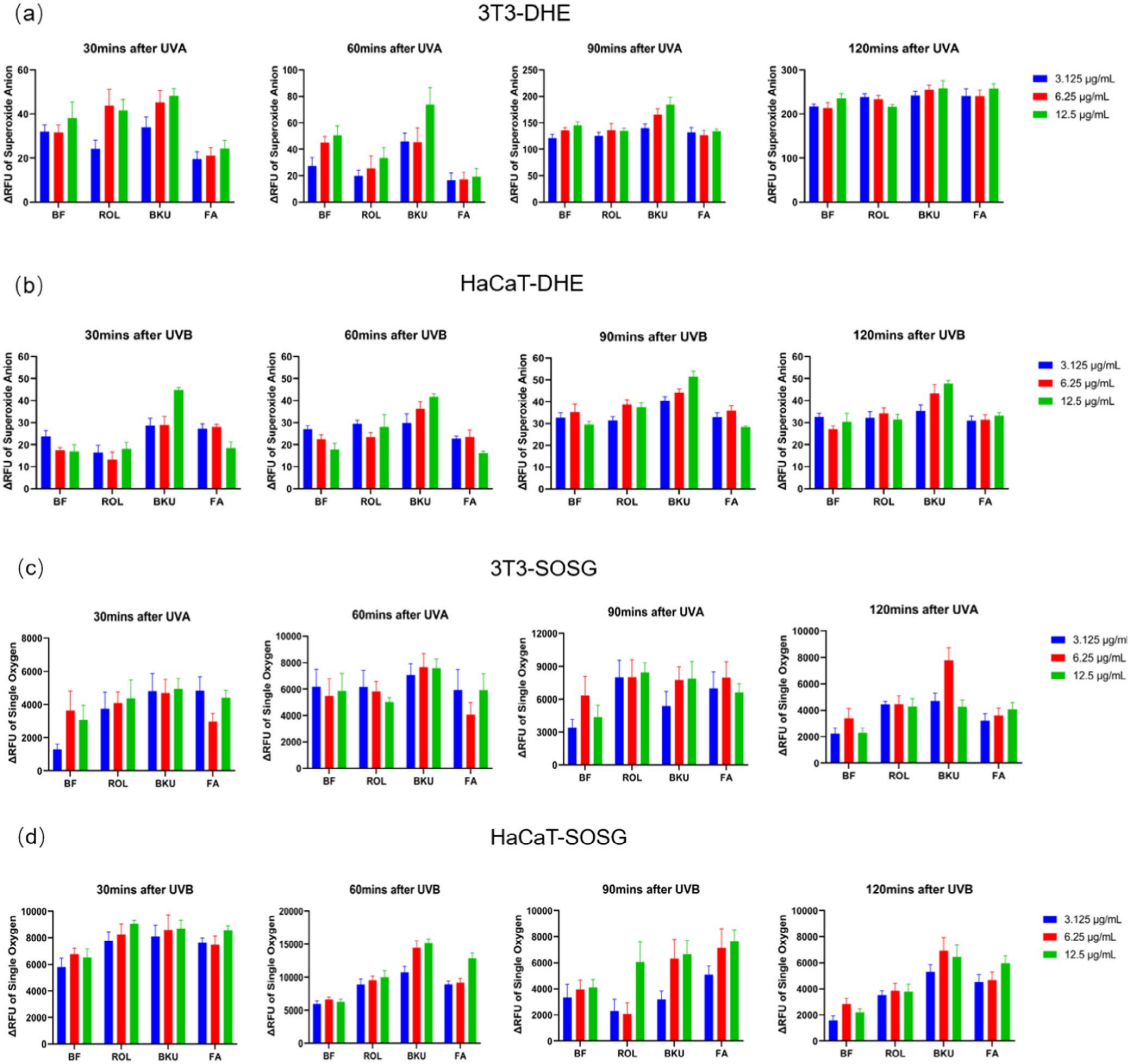
Production level of singlet oxygen and superoxide anion in (a–d) HaCaT or 3T3 upon UVA radiation by BF, ROL, BKU, and FA.

Based on the 3T3‐NRU phototoxicity assay, all four test compounds maintained cell viability above 80% at 12.5 μg/mL. Consequently, this concentration was established as the maximum dose for evaluating the generation of singlet oxygen (^1^O_2_) and superoxide anions (O_2_·^−^) in HaCaT keratinocytes and 3T3 fibroblasts under UV irradiation upon different time points. To facilitate dose–response analysis and validate comparative activity, concentrations of 3.125 and 6.25 μg/mL were also included. As illustrated in Figure 5a,b, BKU elicited the most pronounced increase in superoxide anion production across all three concentrations in both cell models, followed by ROL, BF, and FA. Notably, BF demonstrated the lowest levels of singlet oxygen production (Figure 5c,d), outperforming the other three compounds in both UV‐irradiated 3T3 and HaCaT cells. Collectively, these data indicate that BF consistently induces low levels of UV‐triggered ROS across both epidermal and dermal models, highlighting its superior photostability and minimal phototoxic potential.

### 3.6 Percutaneous Absorption and Skin Retention

**FIGURE 6:**
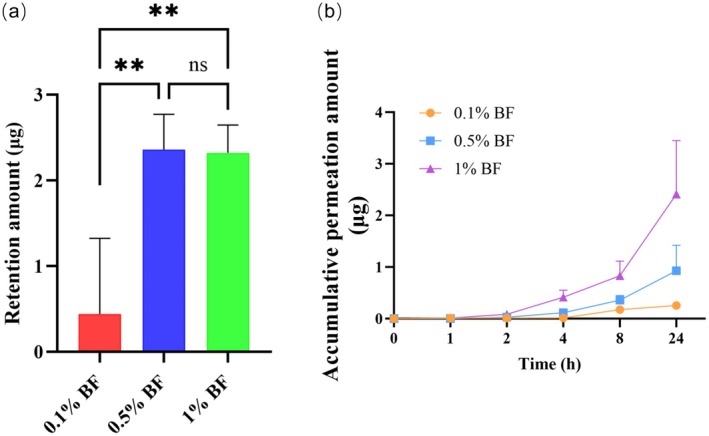
Effects of BF concentration on retention and permeation of BF across Bama miniature pig skin. (a) Retention amount (μg) in the skin at 24 h following application of 0.1%, 0.5%, and 1% BF. (b) Cumulative permeation amount (μg) over 24 h.**p < 0.01.

The in vitro skin permeation of BF exhibited a clear concentration‐dependent profile. As indicated in Figure 6, the 1% BF group demonstrated the highest cumulative permeation at 24 h (19.27 ± 8.34 μg), followed by the 0.5% BF (7.43 ± 3.97 μg) and 0.1% BF (2.04 ± 0.42 μg) groups. Detectable permeation occurred earlier in the 0.5% and 1% BF groups, with cumulative amounts increasing progressively across all cohorts throughout the 24 h period. Analysis of intra‐dermal retention at 24 h revealed that levels in the 1% BF (2.32 ± 0.32 μg) and 0.5% BF (2.36 ± 0.41 μg) groups were significantly higher than those in the 0.1% BF (0.44 ± 0.88 μg). Notably, no significant difference was observed between the 0.5% and 1% BF groups, suggesting that intra‐dermal drug retention may reach a saturation point as the dose increases. Collectively, these data indicate that BF possesses favorable percutaneous absorption, effectively traversing the skin barrier to achieve robust viable epidermal and dermal delivery.

### 3.7 14‐Day Cumulative Irritancy Potential of ROL and BF

The 14‐day cumulative skin irritation potential of the test materials was evaluated by calculating the MCII for each group, with the resulting values and clinical classifications summarized in Table 4. Overall, all tested concentrations of both ROL and BF demonstrated high levels of skin compatibility. Within the ROL groups, a slight concentration‐dependent increase in the irritation index was observed. The 0.3% ROL group recorded an MCII of 0.031 ± 0.093, which resulted in its classification as ‘Slightly irritating’ according to the standardized irritancy scale. In contrast, all concentrations of BF exhibited remarkably low irritancy scores and were classified as “Nonirritating.” Notably, even at the highest concentration of 1% BF maintained a negligible MCII score equivalent to that of the 0.05% concentration. Consequently, all BF test groups and the lower concentrations of ROL (0.05% and 0.1%) were categorized as “Nonirritating.” This indicated a superior tolerance profile for BF even when applied at higher concentrations.

**TABLE 4:** Mean Cumulative Irritancy Index (MCII) and clinical classification of skin irritation for retinol (ROL) and bakuchiol ferulate (BF) after 14 days of cumulative application.

Group	0.05% ROL	0.1% ROL	0.3% ROL	0.05% BF	0.1% BF	0.3% BF	0.5% BF	1% BF
MCII	0.010 ± 0.036	0.014 ± 0.066	0.031 ± 0.093	0.002 ± 0.013	0.010 ± 0.031	0.010 ± 0.052	0.009 ± 0.025	0.002 ± 0.013
Classification of Irritation	Nonirritating	Nonirritating	Slightly irritating	Nonirritating	Nonirritating	Nonirritating	Nonirritating	Nonirritating

### 3.8 Transcriptome Profiling Reveals Retinoid‐Like Gene Expression Patterns of Compound BF

**FIGURE 7:**
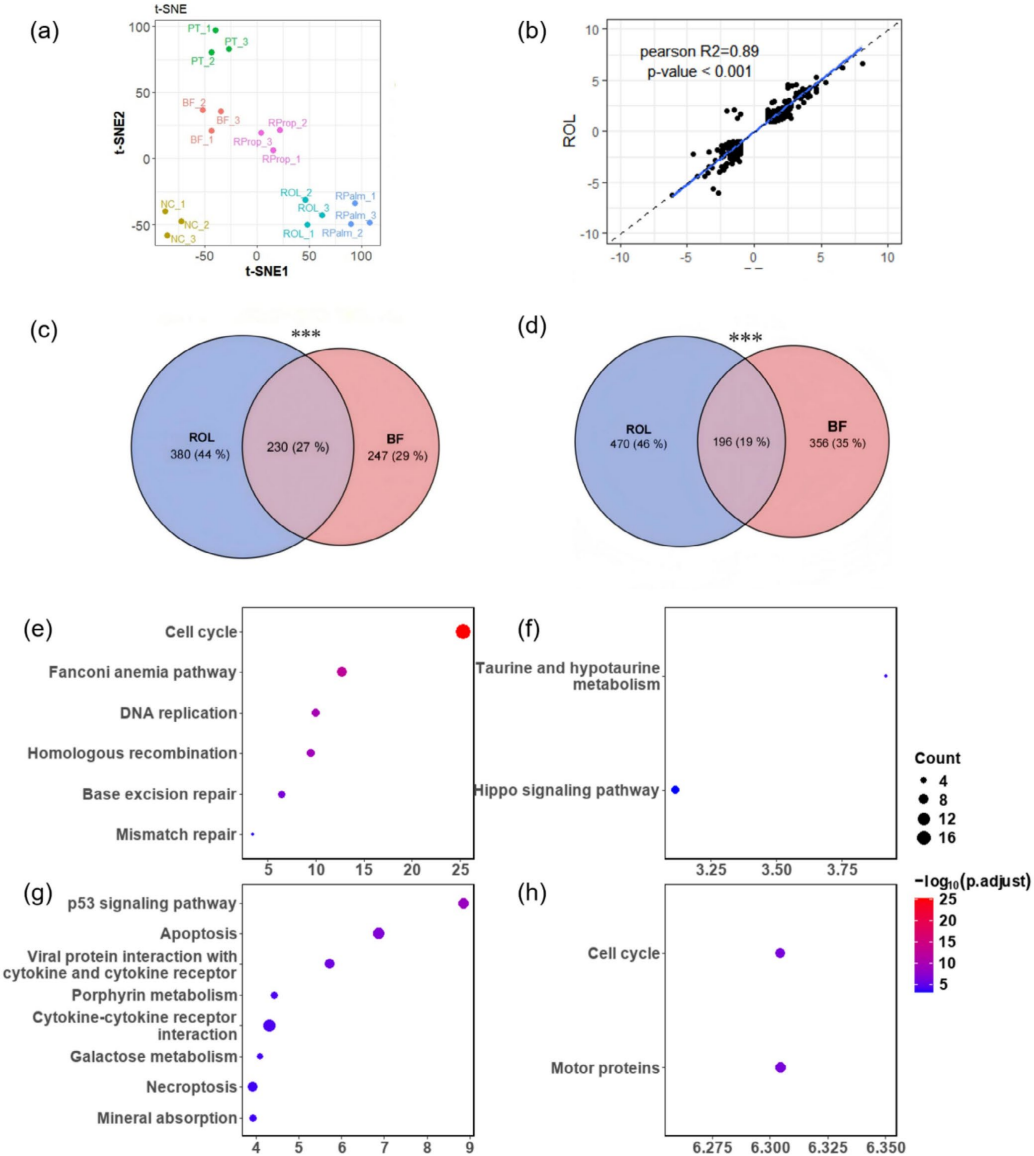
Transcriptome analysis revealed that BF exhibits retinoid‐like characteristics. (a) Principal component analysis (PCA) of the transcriptome in BF, ROL, NC, and retinoid‐like groups. (b) Correlation analysis of fold‐change values in differentially expressed genes (DEGs) shared between BF and ROL. (c, d) Venn diagrams showing differentially upregulated (c) and downregulated (d) DEGs between BF and ROL, with *** indicating significant overlap (Fisher's exact test, *p* < 0.001); (e–h) KEGG pathway analysis of (e) co‐upregulated, (f) co‐downregulated DEGs, (g) ROL‐uniquely upregulated DEGs, and (h) BF‐uniquely upregulated DEGs.

Transcriptome profiling was conducted to characterize the retinoid‐like activity of compound BF. RNA sequencing analysis compared multiple treatment groups in HDF: BF, ROL, phytosterol (PT), retinyl palmitate (RPalm), retinyl propionate (RProp), and vehicle control (NC). The retinoid derivatives RPalm and RProp were included as retinoid analogs, while PT was selected as a plant‐derived compound reported to exhibit retinoid‐like functions. t‐SNE dimensionality reduction revealed distinct clustering patterns, with BF‐treated samples clustering closely with ROL and retinoid analogs while clearly separating from PT and NC groups (Figure 7a), suggesting transcriptional similarity to retinoids.

Transcriptomic correlation analysis demonstrated a strong linear relationship between BF and ROL (Pearson's *R* ^2^ = 0.89, *p* < 0.001; Figure 7b), indicating highly concordant gene expression profiles. Differential expression analysis identified significant overlap between BF and ROL, with 230 co‐upregulated and 196 co‐downregulated genes (Fisher's exact test, *p* < 0.001; Figure 7c,d), further supporting BF's retinoid‐like transcriptional activity.

Pathway enrichment analysis of co‐upregulated genes revealed significant involvement in cell cycle regulation and DNA damage repair pathways, including cell cycle, Fanconi anemia pathway, DNA replication, homologous recombination, and base excision repair (Figure 7e)—processes known to mediate retinoid‐induced skin renewal. Co‐downregulated genes were enriched in taurine/hypotaurine metabolism and Hippo signaling pathway (Figure 7f), both implicated in proliferation and homeostasis regulation.

Notably, ROL‐specific upregulated genes were associated with p53 signaling, apoptosis, cytokine‐cytokine receptor interaction, necroptosis, and mineral absorption (Figure 7g), potentially reflecting ROL‐related adverse effects. In contrast, BF‐specific upregulated genes showed enrichment in motor protein activity and cell cycle processes (Figure 7h), suggesting preserved renewal benefits without concomitant pro‐inflammatory or cytotoxic responses.

These results demonstrate that BF elicits retinoid‐like transcriptional effects highly similar to ROL, while exhibiting a distinct molecular signature that may confer enhanced safety and functionality. However, it must be emphasized that overlapping gene expression patterns do not necessarily translate into comparable biological or clinical effects, and true functional equivalence remains to be fully verified.

## 4 Discussion

Currently, various bioactive compounds including antioxidants are of cosmetics interest. For example, ROL, vitamin C, and vitamin E are all known to benefit human skin cells with antiaging or skin barrier enhanced properties [15, 16]. Despite their therapeutic advantages, the clinical application of these compounds is often limited by biostability concerns and phototoxicity, both of which are highly dependent on the specific dose and formulation used. Therefore, molecular modifications can be made to strengthen their effectiveness. These modifications include improving the chemical stability, enhancing the activity of the pharmacophore, or even modifying their cellular targeting efficiency [17]. Structural modifications that yield a substantial increase in bioactivity, particularly in vivo efficacy, hold the potential to be a breakthrough. Therefore, the pursuit of novel cosmetic ingredients through rational design or structural optimization is essential.

Both FA and BKU are widely used in cosmetology and dermatology for their antioxidant and antiaging activities [5, 18]. Especially, our previous finding revealed that FA in synergy with ROL showed excellent anti‐photoaging capabilities in human keratinocytes [19]. From the chemical structure perspective, FA contains an exposed phenolic hydroxyl group at the C4 position, which may interfere with the esterification between its carboxylic acid group and the hydroxyl group of BKU. Moreover, given the structural similarity of various natural products to FA, the use of FA analogs was considered as alternatives [20, 21]. Based on our preliminary screening of approximately ten FA analogs for stability, irritancy, and efficacy, (E)‐3‐(benzo[d][1,3]dioxol‐5‐yl)acrylic acid was selected as the reactant (data unpublished). Leveraging the structural features of the reactants, the novel compound BF was synthesized via esterification between (2E)‐3‐(1,3‐Benzodioxol‐5‐yl)‐2‐propenoic acid and BKU.

This study aims to employ molecular hybridization from medicinal chemistry to rationally design novel compound BF via molecular modification. The resulting compound BF is expected to give synergistic effects and retain the efficacy of FA and BKU while improving compound stability and reducing cytotoxicity. Indeed, we have verified that BF could serve as the next generation antiaging compound. Our results demonstrated that BF has better overall performance in thermal stability, photostability, and phototoxicity than ROL, BKU, and FA. Cellular data indicated BF could generate less singlet oxygen and superoxide anion upon UV radiation than the other three compounds. Furthermore, the superior chemical stability and reduced cellular toxicity of BF were further validated by our clinical findings in the 14‐day cumulative irritation study. While ROL exhibited a concentration‐dependent increase in irritation—reaching a ‘Slightly irritating’ classification at 0.3%—all concentrations of BF (up to 1.0%) remained within the “Nonirritating” range. These data emphasize the potential of BF for safe and effective use in professional cosmetic applications, particularly for sensitive skin formulations where traditional retinoids may be limited by their irritancy. The enhanced stability of BF can be attributed to key structural modifications: the esterification reaction eliminates the phenol hydroxyl group of BKU, thereby reducing susceptible sites for photo‐oxidation. Furthermore, esterification of the carboxylic acid group of the FA analog alters the electron density of the phenol hydroxyl group via changes in the conjugate system, rendering it less prone to oxidation. Additionally, the adjustment of the overall molecular conjugate system may shift its absorption wavelength, contributing to the improved stability. These data emphasize the potential of BF in safety use during cosmetic applications.

The transcriptomic data further indicated a strong correlation with the ROL‐like biological activity of BF. Co‐upregulated pathways related to the cell cycle and DNA damage repair—including homologous recombination, mismatch repair, and base excision repair—were identified. ROL has been reported to enhance DNA synthesis and regulate the cell cycle by downregulating p21, thereby promoting cell cycle progression [22]. Moreover, retinoids are known to exert protective effects on DNA repair mechanisms [23]. For instance, retinoic acid was shown to reduce single‐strand breaks in liver DNA of p‐dimethylaminoazobenzene (DAB)‐treated rats [24]. Additionally, combined treatment with ROL and RPalm facilitated the repair of UVB‐induced DNA damage via homologous recombination [25]. Based on these findings, we hypothesize that BF may exhibit similar positive effects to those of ROL, presumably by sharing potential molecular targets (e.g., RAR/RXR pathways), but these need further mechanistic studies to validate. Nevertheless, ROL has been documented to cause several adverse effects in cosmetic and dermatologic applications [2]. Consistent with our transcriptomic observations, ROL in higher concentration induces apoptosis and necroptosis by provoking oxidative stress in HDFs [26]. However, these pathways were not enriched in BF‐treated samples, suggesting that BF may not share these negative side effects. Instead, BF distinctly upregulated the motor protein signaling pathway, indicating potential modulation of cell adhesion, migration, and proliferation—processes that could further facilitate skin regeneration and repair [27].

In daily clinical and cosmetic practice, the therapeutic window of topically applied retinoids is heavily restricted by their side‐effect profile, which frequently leads to poor patient compliance or complete treatment discontinuation due to retinoid dermatitis [2]. This clinical challenge is further compounded by shifting global regulatory landscapes; for instance, the European Union's Scientific Committee for Consumer Safety (SCCS) has recently restricted ROL concentrations to a maximum of 0.3% for face products due to systemic safety concerns regarding cumulative Vitamin A intake [14]. The clinical applicability of our findings lies in the potential of BF to fundamentally redefine this safety‐efficacy trade‐off. While traditional retinoids face these strict regulatory and tolerability ceilings at 0.3%, our clinical data demonstrate that BF maintains an entirely nonirritating profile in human subjects even at an elevated concentration of 1.0%, allowing clinicians and formulators to confidently recommend higher active concentrations without compromising skin barrier safety. This drastically simplifies daily antiaging skincare regimens, effectively enhancing long‐term user adherence and ensuring a highly predictable, irritation‐free clinical outcome. Despite revealing the promising activity of BF, we also acknowledge that several limitations are presented in this study. In our stability assay, BF, ROL, and BKU were formulated in isononyl isononanoate; FA was dissolved in dipropylene glycol due to its specific solubility requirements. The resulting variation in solvent polarity should be considered a potential confounding factor when comparing FA to the lipophilic analytes. Moreover, the detailed mechanisms underlying its function in skin aging remain to be elucidated in this study. Further study can be done in the future to elucidate the exact molecular targets and metabolic profile of BF by molecular docking and receptor activation assays in tackling skin aging. Translating these molecular similarities into comparable clinical efficacy requires further phenotypic validation. While the current study establishes the stability and clinical safety of BF, further research is required to evaluate its long‐term functional efficacy. Future in vivo studies or clinical trials should focus on longitudinal assessments of its antiaging performance—specifically regarding collagen synthesis and wrinkle reduction—to fully characterize its bioactivity in skin.

## 5 Conclusions

Collectively, our findings demonstrate that BF exhibits a transcriptomic profile highly correlated with a potent ROL‐like compound that retains beneficial biological pathways with less adverse effects. Through molecular hybridization, BF exhibits superior photostability and reduced phototoxicity compared to ROL, BKU, and FA, rendering it suitable for next‐generation antiaging formulations. Our findings provide a robust safety foundation for the clinical application of BF. By integrating the structural stability of BKU with the antioxidant capacity of FA, BF represents a novel retinoid‐like molecule with enhanced durability and bioactivity. At the transcriptomic level, BF closely recapitulates the beneficial gene expression programs of ROL, particularly pathways related to cell cycle progression and DNA damage repair, while avoiding enrichment of proapoptotic and proinflammatory pathways. These attributes position BF as a promising and well‐tolerated alternative to ROL in cosmetic and dermatological applications, particularly for individuals with sensitive skin or long‐term use demands.

## Author Contributions

**Jiangming Zhong** and **Peng Shu:** conceptualization, supervision, project administration. **Jiangming Zhong**, **Yizhen Yan**, **Nan Zhao**, **Xinyu Cao**, **Yuting Liang**, **Zhiwei Li**, **Qiaoyuan Liu**, **Meixin Lu**, and **Jinjing Bao:** methodology. **Yizhen Yan**, **Nan Zhao**, **Xinyu Cao**, and **Yuting Liang:** software. **Jiangming Zhong**, **Yizhen Yan**, **Nan Zhao**, **Xinyu Cao**, **Yuting Liang**, **Qi Zhou**, and **Jinjing Bao:** validation. **Jiangming Zhong**, **Yizhen Yan**, **Nan Zhao**, **Xinyu Cao**, **Yuting Liang**, **Jinjing Bao**, **Zhiwei Li**, **Qing Liu**, **Meixin Lu**, and **Bin Shuai:** formal analysis. **Jiangming Zhong**, **Yizhen Yan**, **Nan Zhao**, **Xinyu Cao**, **Yuting Liang**, and **Jinjing Bao:** investigation. **Jiangming Zhong**, **Yizhen Yan**, **Nan Zhao**, **Xinyu Cao**, **Yuting Liang**, and **Jinjing Bao:** data curation. **Jiangming Zhong** and **Yizhen Yan:** writing – original draft preparation. **Nan Zhao**, **Xinyu Cao**, **Qi Zhou**, and **Rong Hu:** writing – review and editing. **Yuting Liang**, **Nan Zhao**, and **Xinyu Cao:** visualization. **Jiangming Zhong and Peng Shu:** supervision and project administration.

## Conflicts of Interest

The authors declare no conflicts of interest.

## Supporting information


**Figure S1:** jocd71067‐sup‐0001‐FigureS1‐S2.docx. ^1^H NMR spectrum of compound BF.
**Figure S2:** ^13^C NMR spectrum of compound BF.

## Data Availability

The data that support the findings of this study are available from the corresponding author upon reasonable request.
