# Supplementary material for: Bakuchiol Ferulate: A Novel Functional Retinol Analog With Enhanced Photostability and Reduced Phototoxicity for Cosmetic Applications
Source: J Cosmet Dermatol. 2026 Jul 16;25(7):e71067. doi: 10.1111/jocd.71067 (PMC13373746; doi:10.1111/jocd.71067)
Supplement: Supplementary file 1 — Figure S1: jocd71067‐sup‐0001‐FigureS1‐S2.docx. 1H NMR spectrum of compound BF. Figure S2: 13C NMR spectrum of compound BF. [file JOCD-25-e71067-s001.docx]

**Structural Characterization of Compound BF**


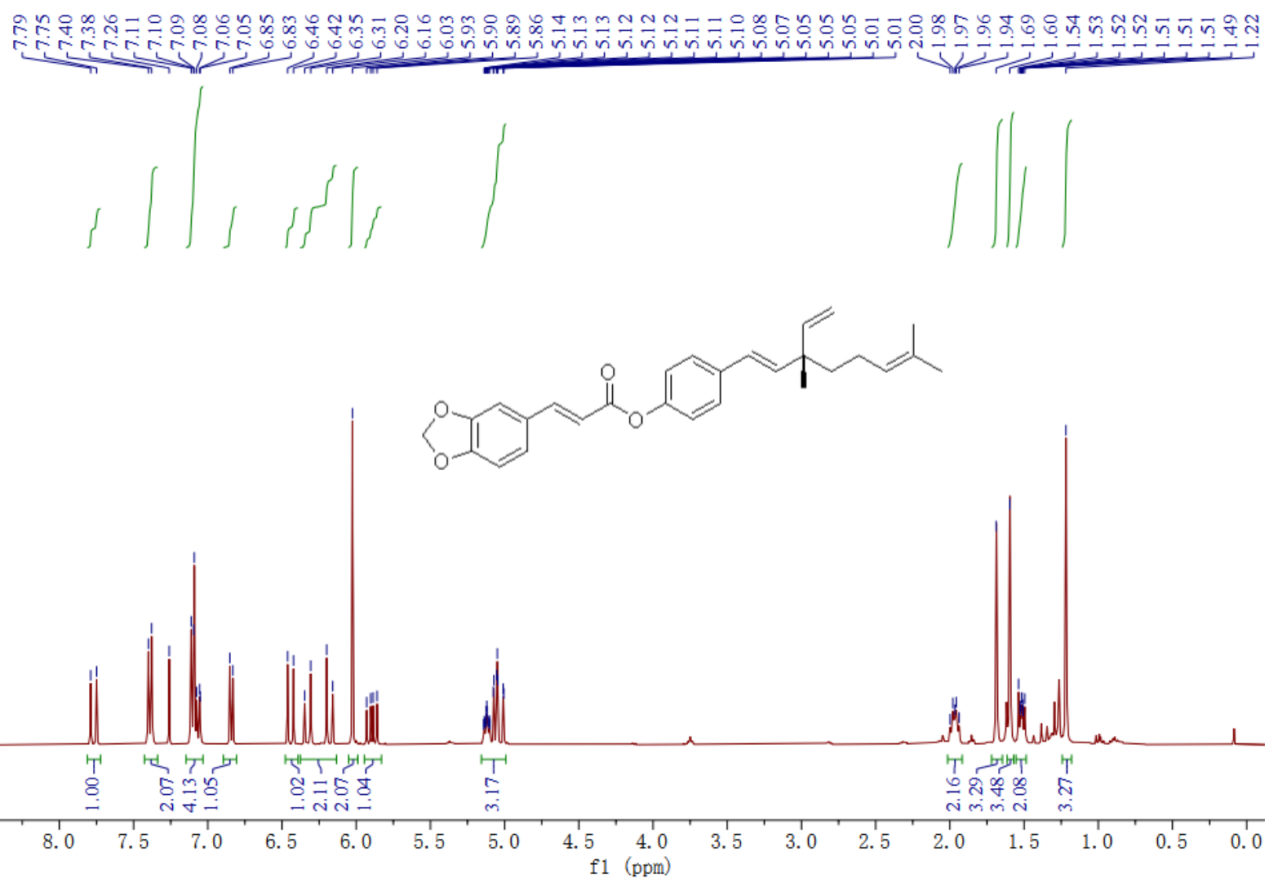


Supplementary Figure 1. ^1^H NMR spectrum of compound BF.


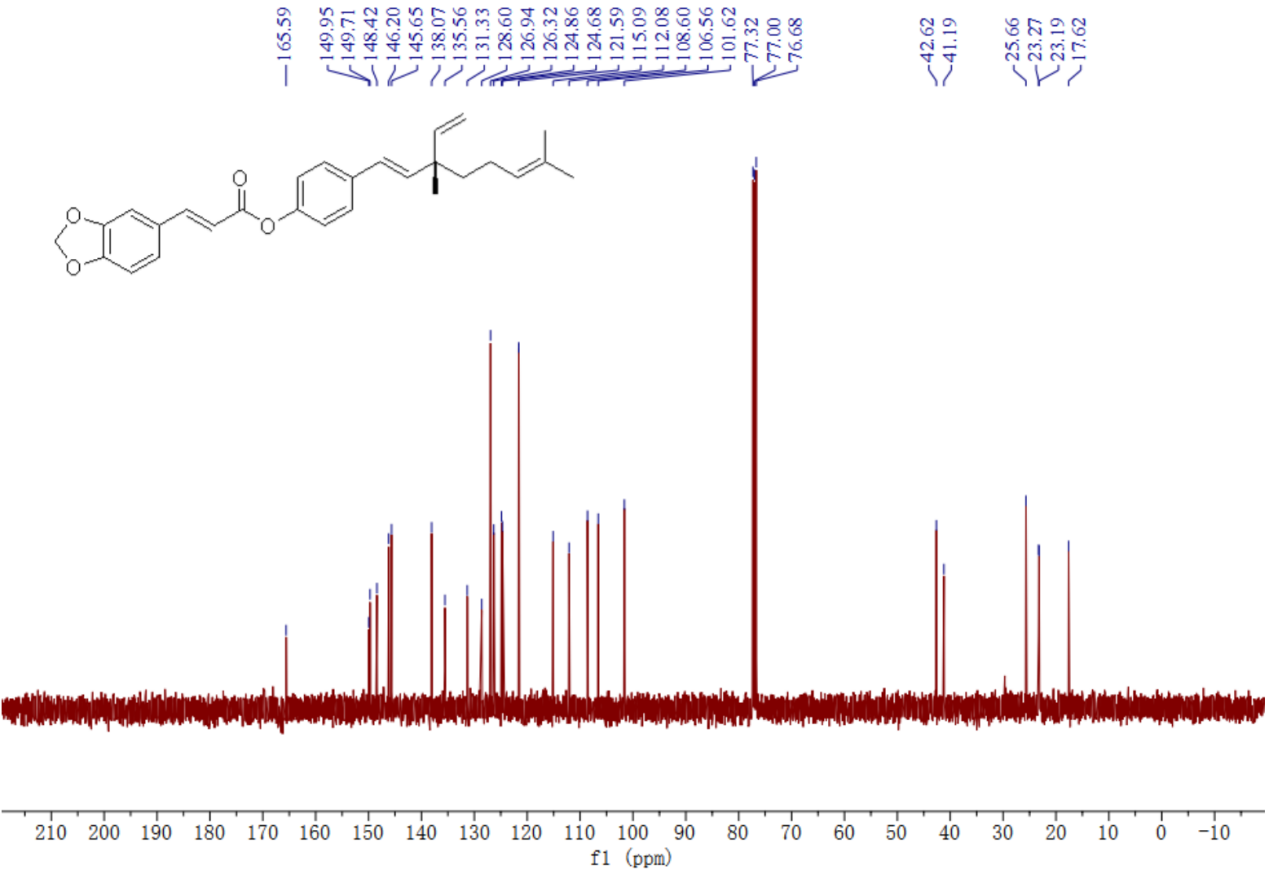


Supplementary Figure 2. ^13^C NMR spectrum of compound BF.
